# The Impact of Age and Gender on Cervical Vestibular-Evoked Myogenic Potentials Using 500 Hz Tone Bursts Stimuli: A Cross-Sectional Study

**DOI:** 10.7759/cureus.84142

**Published:** 2025-05-15

**Authors:** Saumya Pandey, Sangeeta Gupta, Rama S Rath, Gaurav Gupta

**Affiliations:** 1 Physiology, All India Institute of Medical Sciences (AIIMS) Gorakhpur, Gorakhpur, IND; 2 Community Medicine, All India Institute of Medical Sciences (AIIMS) Gorakhpur, Gorakhpur, IND; 3 General Surgery, All India Institute of Medical Sciences (AIIMS) Gorakhpur, Gorakhpur, IND

**Keywords:** age, amplitude, amplitude asymmetry, cvemp, gender, latency, sternocleidomastoid, threshold, vestibular evoked myogenic potentials

## Abstract

Background and objective

Cervical vestibular-evoked myogenic potential (cVEMP) tests have attracted significant interest as clinical measures of vestibular function recently. A detailed knowledge of the potential individual factors influencing the cVEMP response is, however, essential for the reliability of the test. Hence, we aimed to analyze the trends of different cVEMP parameters with regard to changes in age and gender, under standard recording conditions.

Methods

cVEMP was conducted on 60 participants aged 18-60 years during the period from April 2023 to March 2024. cVEMP parameters, including response rates, amplitude, interaural amplitude asymmetry, threshold, threshold asymmetry, and latencies, were investigated for the impact of aging and gender differences. One-way ANOVA and Kruskal-Wallis tests were performed for the comparison between the age groups, while unpaired t-test and Kruskal-Wallis test were employed to find the gender differences in cVEMP parameters. Pearson correlation analysis was performed to assess the relationship between age and cVEMP parameters. Statistical significance was set at a p-value of 0.05.

Results

cVEMP response rate was 100 % in all the age groups. Significant differences were found in terms of p13-n23 amplitude and threshold (both right and left ear) between the age groups (p<0.001) (Kruskal-Wallis test). A significant negative correlation was found between age and both uncorrected and corrected amplitude in both the right ear (r = -0.73, p<0.001) and the left ear (r = -0.75, p<0.001). A significant positive correlation was observed between age and thresholds in both the right ear (r = 0.66, p<0.001) and the left ear (r = 0.75, p<0.001). There were no significant variations regarding p13 and n23 latencies, interaural amplitude asymmetry, and threshold asymmetry among the age groups (p>0.05) (one-way ANOVA) (Kruskal-Wallis test). Gender did not demonstrate variations in the cVEMP parameters tested (p>0.05) (unpaired t test and Kruskal-Wallis test).

Conclusions

cVEMP had an absolute detection rate in the age groups studied. However, a remarkable age-related decline in p13-n23 amplitude (corrected and uncorrected) and the threshold values highlights the importance of effective consideration of changes in age for optimizing the clinical evaluation. p13 and n23 latencies, and amplitude asymmetry were relatively stable with increasing age. The findings related to cVEMP evoked with 500 Hz short tone burst stimuli in adults (aged 18-60 years) can be utilized in the regional clinical settings.

## Introduction

Recent years have witnessed a significant surge in neurophysiological vestibular research, which has broadened the range of currently available vestibular tests. With the advent of cervical and ocular vestibular-evoked myogenic potentials (cVEMP and oVEMP), it is now feasible to investigate the sacculo-spinal and utriculo-ocular pathways in regular clinical settings. Otolithic function and its afferent pathways are frequently assessed by VEMPs as part of electrophysiological investigations. The test offers a simple, reproducible tool for subjects without conductive hearing loss. While oVEMP evaluates utricular function and the superior vestibular nerve, cVEMP investigates saccular function and the inferior vestibular nerve integrity. cVEMPs are elicited by vestibulo-collic reflexes arising from the saccule that travel through the inferior vestibular nerve. On the other hand, oVEMPs are recorded based on the vestibulo-ocular reflex from the utricle and superior vestibular nerve [1-3]. VEMP enables the evaluation of vertigo and supports the diagnosis of many clinical conditions including superior semicircular canal dehiscence syndrome, vestibular neuritis, vestibular schwannoma, and Menière's disease, when used in conjunction with Videonystagmography and Video Head Impulse Test. cVEMP tests have gained considerable recognition and relatively greater acceptance as clinical tests of vestibular function [4].

Nonetheless, an extensive knowledge of the variables that could affect the recorded responses is necessary to ensure reliable VEMP tests. The technical factors that require consideration include the type of stimuli used to elicit the responses, patient positioning, electrode montages, etc. The most commonly employed methods for cVEMPs in the clinical context are air-conducted (AC) stimuli, which can induce robust saccular responses for cVEMPs except when conductive losses are present [5]. Most studies use 500 Hz tone burst AC stimuli for cVEMPs. However, the tuning of the VEMP has been reported to shift to a higher frequency (750 Hz) in older adults (≥60 years) [6]. Regarding the type of AC stimuli to elicit the responses, despite 100% response rates for both short-tone bursts (STBs) and click stimuli, STBs have been reported to be the ideal stimulus for evoking the cVEMP responses [7,8]. When the patient positioning is taken into account, larger amplitudes have been reported in sitting positions for cVEMP recording [7,9].

Even though standard stimuli and recording settings have been employed for the tests, certain individual aspects are known to impact the VEMP test, such as activity of the sternocleidomastoid muscle (SCM), age, and gender [9-11]. Studies have shown evidence depicting the alteration of VEMP parameters with increasing age, which may have an impact on the diagnosis of vestibular dysfunction [10-12]. Furthermore, a crucial aspect in correctly interpreting the VEMP test involves keeping constant attention on the SCM's contraction state [13]. The strength of SCM's contraction is important as the amplitude is found to be larger with stronger contraction. Hence, the SCM contraction should be optimum and bilaterally uniform in cVEMP recording and measurement. The use of corrected amplitude [obtained by dividing the cVEMP amplitude by electromyography (EMG) estimate of muscle contraction] minimizes the influence of SCM contractions on the amplitudes, corrects the contraction difference, and provides corrected interaural amplitude asymmetry ratios [9].

Another individual attribute that may influence the cVEMP parameters is gender, which has very sparsely been investigated in the published literature [14]. Moreover, other factors, such as ethnicity, genetics, and anatomical differences, may also impact VEMP responses, in addition to aging [10]. The above-mentioned findings highlight the notable role of individual attributes in cVEMP measurements. In light of this, the objective of this study was to investigate the impact of age and gender on cVEMP test parameters by ensuring an optimum and bilaterally uniform level of SCM contraction in a sample of healthy participants, representing both the young and the late middle age groups.

## Materials and methods

This study involved 60 participants and spanned the period from April 2023 to March 2024. It adopted an analytical cross-sectional study design. The study was conducted at the Neurophysiology Laboratory, Department of Physiology, All India Institute of Medical Sciences (AIIMS), Gorakhpur. The sample size was determined based on a power of 80% and a significance level of 5% using the one-way analysis of variance (ANOVA) (Stata version 12) for the participants in three different age groups of[12]. Assuming a non-response rate of 10%, the minimum sample size required was 10 participants for each age group. Prior approval was obtained from the Institute’s Human Ethics Committee (ref no: IHEC/AIIMS-GKP/BMR/123/2023, dated 25.03.2023).

Inclusion criteria of the study were as follows: subjects aged 18-60 years, with normal otological and vestibular examination. Subjects with a history of otological, vestibular, neurological, or neuromuscular disorders, those with a history of cerebral trauma, or those who could not perform neck movements were excluded. The participants recruited were the healthy attendants of the patients visiting the hospital outpatient department (OPD) and Neurophysiology Laboratory at the institute. Participants were assessed for eligibility by taking a detailed history of the presence of neuropathy, myopathy, or previous cerebral trauma, with the help of a questionnaire [15]. All participants underwent ABER (auditory brainstem-evoked responses) before the study to exclude otological dysfunctions. A detailed explanation of the duration and type of the research was provided, and informed written consent was obtained before the study. Appropriate instructions regarding the test procedure were provided before the study.

cVEMP recording

The equipment used for recording the cVEMP test was Neuro-MEP 8 (8-channel NCS, EMG, and multi-modality EP system). Neuro-MEP.netω electromyography software (M/S Neurosoft Ltd, Ivanovo, Russia) was employed for recording surface EMG. The procedure was undertaken in a sound-attenuated room with a uniform temperature. The skin was prepared appropriately before the application of surface electrodes.

Procedure

The acoustic stimulus (500 Hz AC STB) (95 dB nHL, rarefaction polarity, rise/fall time 0 ms, plateau 2.67 ms, stimulation rate 5/s) was presented monoaurally by way of headphones (TA-01). The response window was set within 50 milliseconds (ms) and averaged over 200 stimuli for each run. The signal was band-pass filtered between 30 Hz and 2000 Hz. Amplifier gain was 2500-5000. A reference electrode was placed at the sternoclavicular junction, active at the midpoint of SCM, and ground at the forehead (Fpz) (10-20 system). 

Participants were made to sit in the upright position. Appropriate movements of the neck (SCM muscle contraction) to turn their head contralateral to the ear receiving the acoustic stimulus were performed by the subjects. Surface EMG was recorded during the VEMP recording (gain: around 2000) with a sampling rate of approximately 2-5 kHz. The filter setting was 1-5 Hz (high pass, low cut) to approximately 200 Hz to 1000 Hz (low pass, high cut). The background contraction was measured from the same electrode pair used to record the cVEMP. Muscle contraction was assessed by viewing the EMG during muscle contraction as a visual guide to control the patient’s effort and to ensure optimum contraction. Also, the background EMG estimate was used to calculate an amplitude ratio by dividing the VEMP amplitude by the estimate of muscle contraction (corrected amplitude). 

The procedure employed was per the guidelines reported by Papathanasiou et al. (2014) (International guidelines for the clinical application of cervical vestibular evoked myogenic potentials: An expert consensus report) [16]. cVEMP variables measured were P1 and N1 (P13 and N23) latencies, threshold stimulus (the lowest amplitude sound stimulus that still elicits a reproducible cVEMP response), interaural threshold asymmetry, P13-N23 peak to peak amplitude, interaural amplitude asymmetry [(right - left)/(right + left)], and interaural amplitude asymmetry ratio [(right - left)/(right + left) x 100] (Jongkees formula). Corrected amplitudes were obtained by dividing the amplitude values by the EMG estimate of SCM muscle contraction. 

Statistical analysis

Normality of distribution of the data was assessed using the Shapiro-Wilk test. Summary statistics of normally distributed data were expressed as mean ± standard deviation (SD) or as median (25th percentile - 75th percentile). One-way ANOVA and Kruskal-Wallis test were performed to identify the differences between the age groups, while unpaired t-test and Kruskal-Wallis test were used to find the gender differences. Correlation analysis was done using Pearson/Spearman correlation. The level of statistical significance was set at 0.05. All the analyses and graphical visualizations were performed using the statistical software, Stata version 12 (StataCorp LLC, College Station, TX).

## Results

Sixty subjects aged 18-60 years underwent cVEMP recording (mean age: 36.25 ± 12.07 years). Of these, 30 (50%) were men and 30 (50%) were women. cVEMPs were bilaterally recordable in all the participants. The participants were categorized into three age groups: early adult/young adults: 18-34 years (Group 1), early middle age: 35-44 years (Group 2), and late middle age: 45-60 years (Group 3). Twenty-nine subjects constituted the young/early adult age group, while early and late middle age groups comprised 15 and 16 subjects, respectively. The cVEMP parameters and their comparison between the three age groups are depicted in Table 1. No significant differences were observed for p13 and n23 latencies for both right and left ears among the age groups (p>0.05) (one-way ANOVA) (Table 1). Also, interaural amplitude asymmetry and threshold asymmetry were not significantly altered among the three age groups (p>0.05) (Kruskal-Wallis test).

**Table 1:** Comparison of cVEMP parameters in different age groups ^a^F-value (one-way ANOVA). ^b^Chi-square value (Kruskal-Wallis test) ^*^P-value=1.253 × 10^-9^, post-hoc Dunn's test (multiple comparison): comparison within Groups 1 and 2, p-value=9.39 x 10^-5^; Groups 1 and 3, p-value=9.6 x 10^-10^ ^**^P-value=5.15 x 10^-9^, post-hoc Dunn's test (multiple comparison): comparison within Groups 1 and 2, p-value=0.0007; Groups 1 and 3, p-value=1.7 x 10^-9^; Groups 2 and 3: p=0.02) ^***^P-value=4.86 x 10^-9^, post-hoc Dunn's test (multiple comparison): comparison within Groups 1 and 2, p-value=1.3 x10^-5^; Groups 1 and 3, p-value=1.77 x 10^-8^ ^****^P-value=1.05 x 10^-8^, post-hoc Dunn's test (multiple comparison): comparison within Groups 1 and 2, p-value=0.0002; Groups 1 and 3, p-value=6.9 x 10^-9^ ^*****^P-value=1.5 x 10^-8^, post-hoc Dunn's test (multiple comparison): comparison within Groups 1 and 2, p-value=0.00006; Groups 1 and 3, p-value=2.33 x 10^-8^ ^*****^P-value=2.42 x 10-^9^, post-hoc Dunn's test (multiple comparison): comparison within Groups 1 and 2, p-value=0.00003; Groups 1 and 3, p-value=3.83 x 10^-9^ ANOVA: analysis of variance; cVEMP: cervical vestibular-evoked myogenic potentials; dB nHL: decibels normalized hearing level; IQR: interquartile range; ms: milliseconds; SD: standard deviation; µv: microvolts

cVEMP parameters	Age groups, years			F-value/chi-square value	P-value
	18-34 (Group 1)	35-44 (Group 2)	45-60 (Group 3)		
Right ear p13 latency, ms, mean ±SD	13.87 ±1.32	13.57 ±1.38	13.96 ±1.58	0.33^a^	0.72
Right ear n23 latency, ms, mean ±SD	22.55 ±1.21	22.6 ±1.81	23.37 ±1.62	1.66^a^	0.19
Left ear p13 latency, ms, mean ±SD	14.01 ±1.03	13.25 ±1.44	13.38 ±11.15	2.63^a^	0.08
Left ear n23 latency, ms, mean ±SD	22.29 ±1.19	22.12 ±1.69	22.63 ±1.63	0.49^a^	0.61
Right ear uncorrected amplitude, µv, median (IQR)	129 (104.35-166.5)	52 (35-77.5)	33.35 (27.95-36.2)	40.99^b^	<0.001^*^
Right ear corrected amplitude, µv, median (IQR)	0.52 (0.43-0.76)	0.31 (0.23-0.43)	0.18 (0.16-0.23)	38.16^b^	<0.001^**^
Left ear uncorrected amplitude, µv, median (IQR)	145 (89.9-171.5)	39.5 (31.8-55.1)	33.2 (26.65-46.5)	38.28^b^	<0.001^***^
Left ear corrected amplitude, µv, median (IQR)	0.58 (0.43-0.72)	0.26 (0.19-0.36)	0.19 (0.18-0.24)	36.75^b^	<0.001^****^
Right ear threshold, dB nHL, median (IQR)	80 (77-81 )	83 (81-84)	84 (83-85)	35.99^b^	<0.001^*****^
Left ear threshold, dB nHL, median (IQR)	80 (79-81)	83 (83-84)	84 (84-85)	39.68^b^	<0.001^******^
Amplitude asymmetry (uncorrected), µv, median (IQR)	0.02 (–0.095-0.11)	0.12 (–0.04-0.21)	–0.02 (–0.16-0.09)	3.77^b^	0.15
Amplitude asymmetry (corrected), µv, median (IQR)	0 (–0.085-0.07)	0.09 (–0.06-0.15)	–0.03 (–0.15-0.08)	3.11^b^	0.21
Threshold asymmetry, dB nHL, median (IQR)	–1 (–2-0.5)	0 (–1-1)	0 (–1-0.5)	1.59^b^	0.45

**Figure 1:**
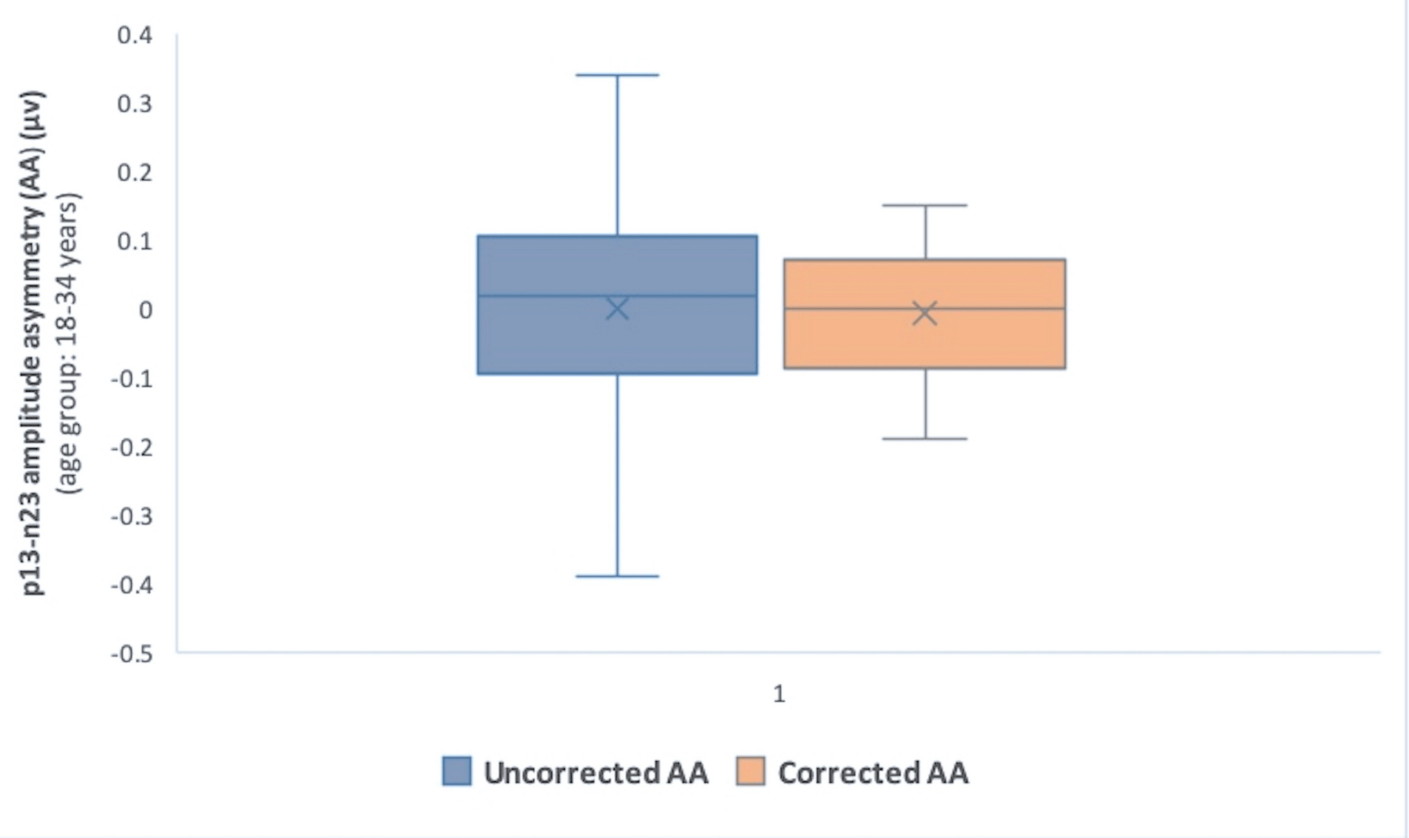
Comparison of corrected and uncorrected interaural p13-n23 AA in Group 1 (18-34 years) P-value=0.88 (Wilcoxon signed-rank test) AA: amplitude asymmetry; µv: microvolts

**Figure 2:**
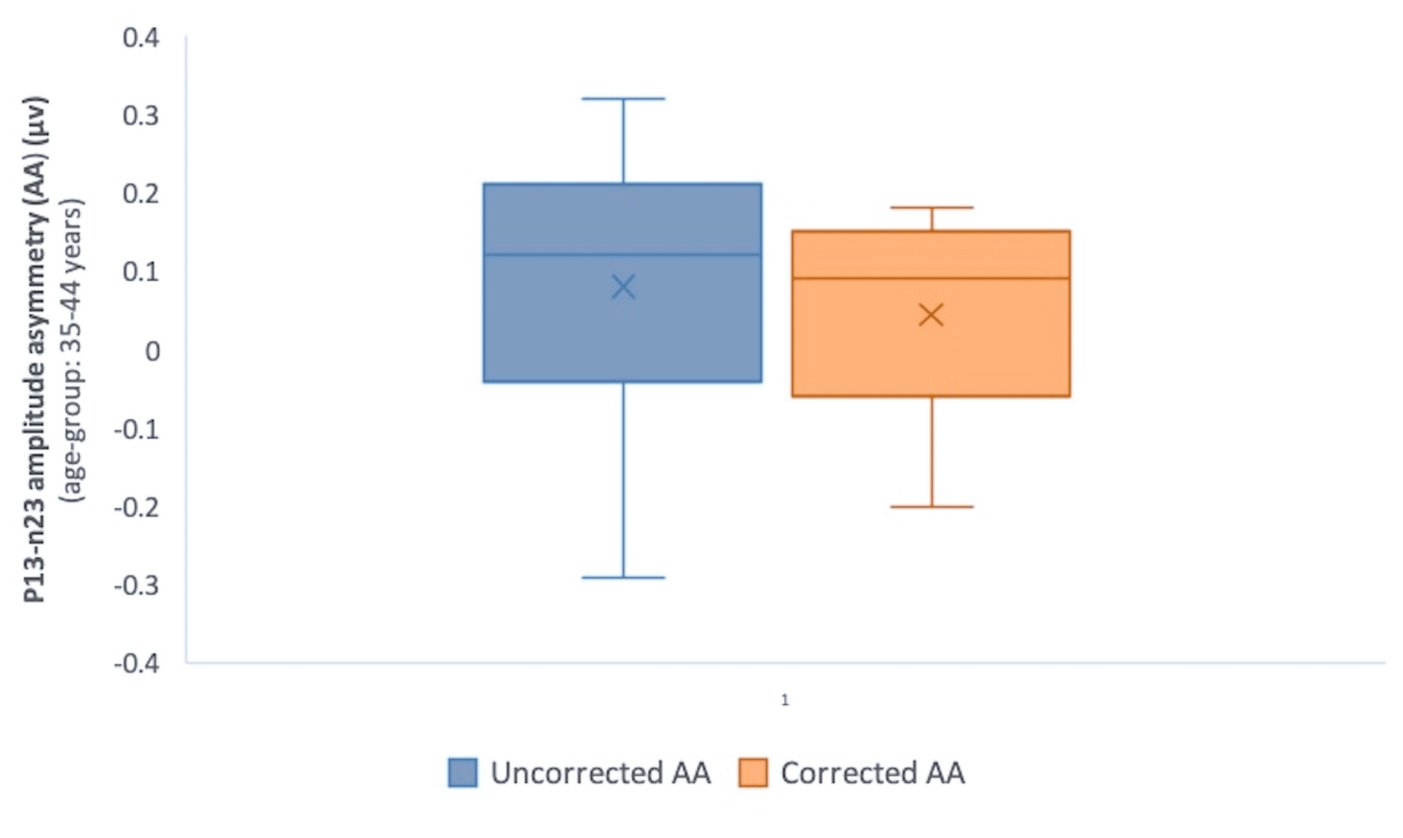
Comparison of corrected and uncorrected interaural p13-n23 AA in Group 2 (35-44 years) P-value=0.22 (Wilcoxon signed-rank test) AA: amplitude asymmetry; µv: microvolts

**Figure 3:**
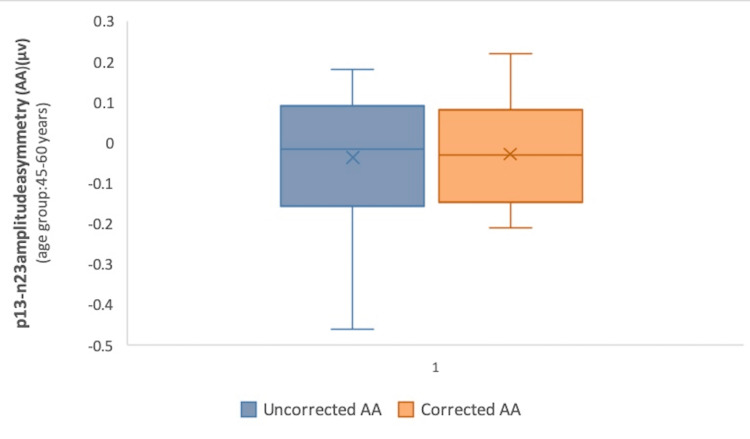
Comparison of corrected and uncorrected interaural p13-n23 AA in Group 3 (45-60 years) P-value=0.95 (Wilcoxon signed-rank test) AA: amplitude asymmetry; µv: microvolts

However, interaural amplitude asymmetry (AA) computed with corrected amplitude values decreased in all the age groups as compared to those calculated with uncorrected amplitude (Table 2) (Figures 1, 2, 3). The difference, however, was not statistically significant (p>0.05) (Wilcoxon signed-rank test).

**Table 2:** Corrected and uncorrected interaural p13-n23 AA in different age groups Corrected AA values decreased in comparison with uncorrected AA in all the age groups (p>0.05) (Wilcoxon signed-rank test) AA: amplitude asymmetry; IQR: interquartile range; µv: microvolts

Age group, years	Uncorrected AA, µv, median (IQR)	Corrected AA, µv, median (IQR)	P-value
18-34	0.02 (–0.095-0.11)	0 (–0.085-0.07)	0.88
35-44	0.12 (–0.04-0.21)	0.09 (–0.06-0.15)	0.22
45-60	–0.02 (–0.16-0.09)	–0.03 (–0.15-0.08)	0.95
18-60 (total)	0.045 (–0.11-0.14)	0.025 (–0.11-0.1)	0.47

Right ear and left ear amplitude, as well as right and left ear threshold, revealed significant differences between the age groups (p<0.001) (Kruskal-Wallis test) (Table 1). The post-hoc Dunn's test using a Bonferroni correction revealed significant differences between Groups 1 and 2 and within Groups 1 and 3 (p<0.001) for the aforementioned parameters.

**Figure 4:**
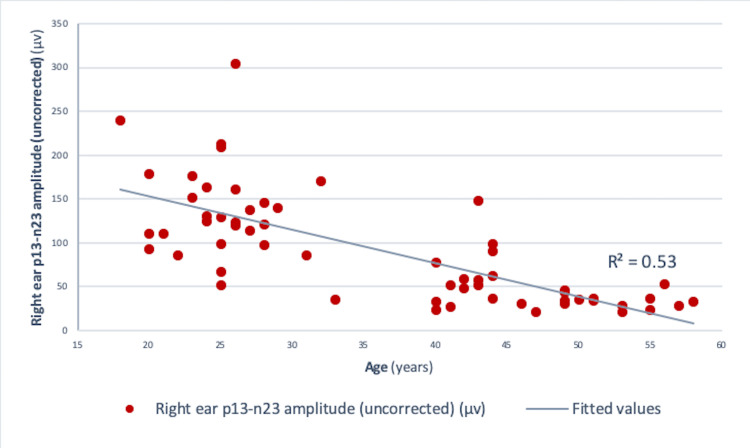
Scatter plot showing the relationship between age (in years) and right ear p13-n23 amplitude (uncorrected) (µv) Significant negative correlation observed between the age and right ear uncorrected amplitude (r = -0.73, r² = 0.53, p<0.001) (Pearson correlation analysis) µv: microvolts

**Figure 5:**
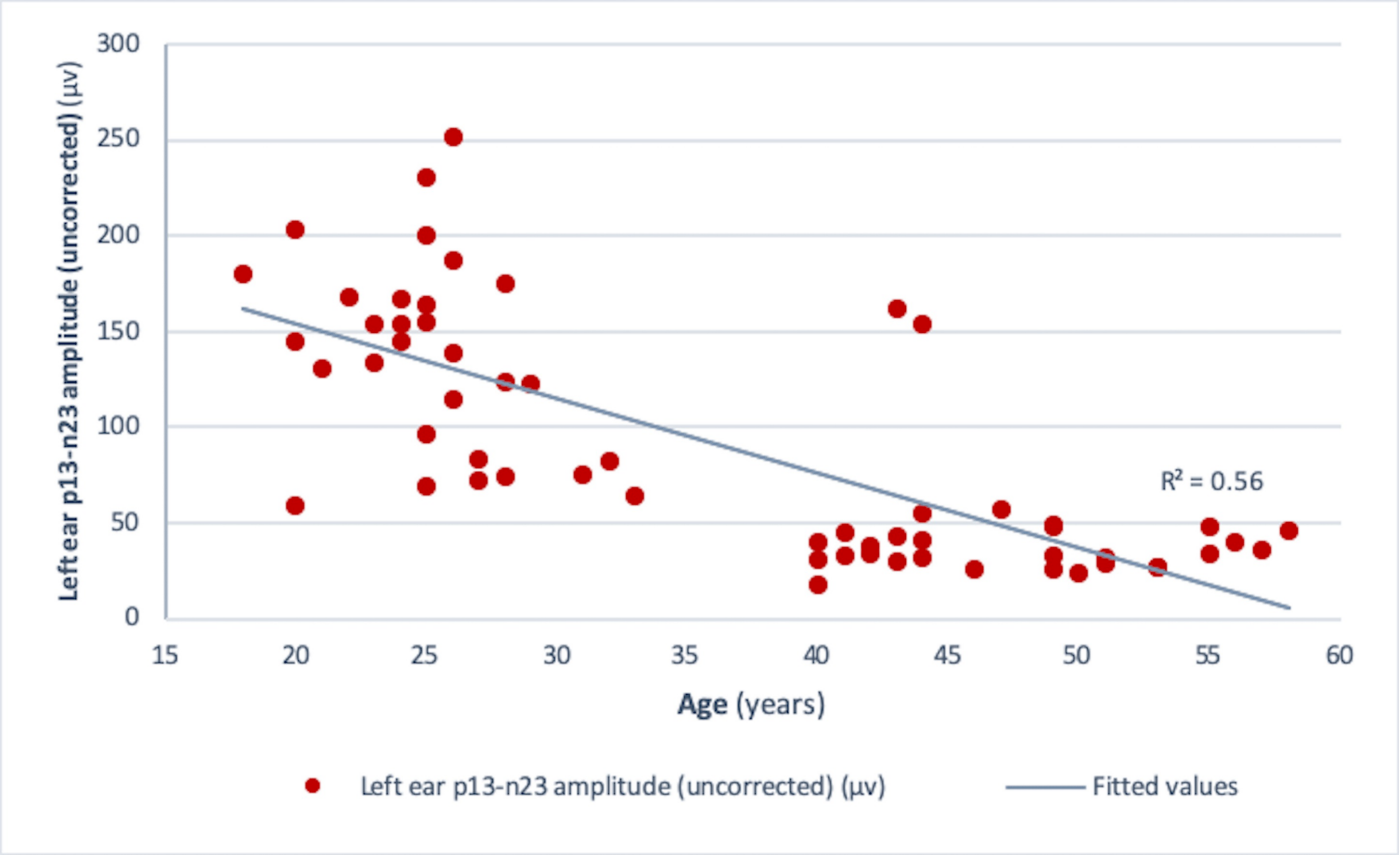
Scatter plot showing the relationship between age (in years) and left ear p13-n23 amplitude (uncorrected) (µv) Significant negative correlation observed between the age and left ear uncorrected amplitude (r = -0.75, r² = 0.56, p<0.001) (Pearson correlation analysis) µv: microvolts

**Figure 6:**
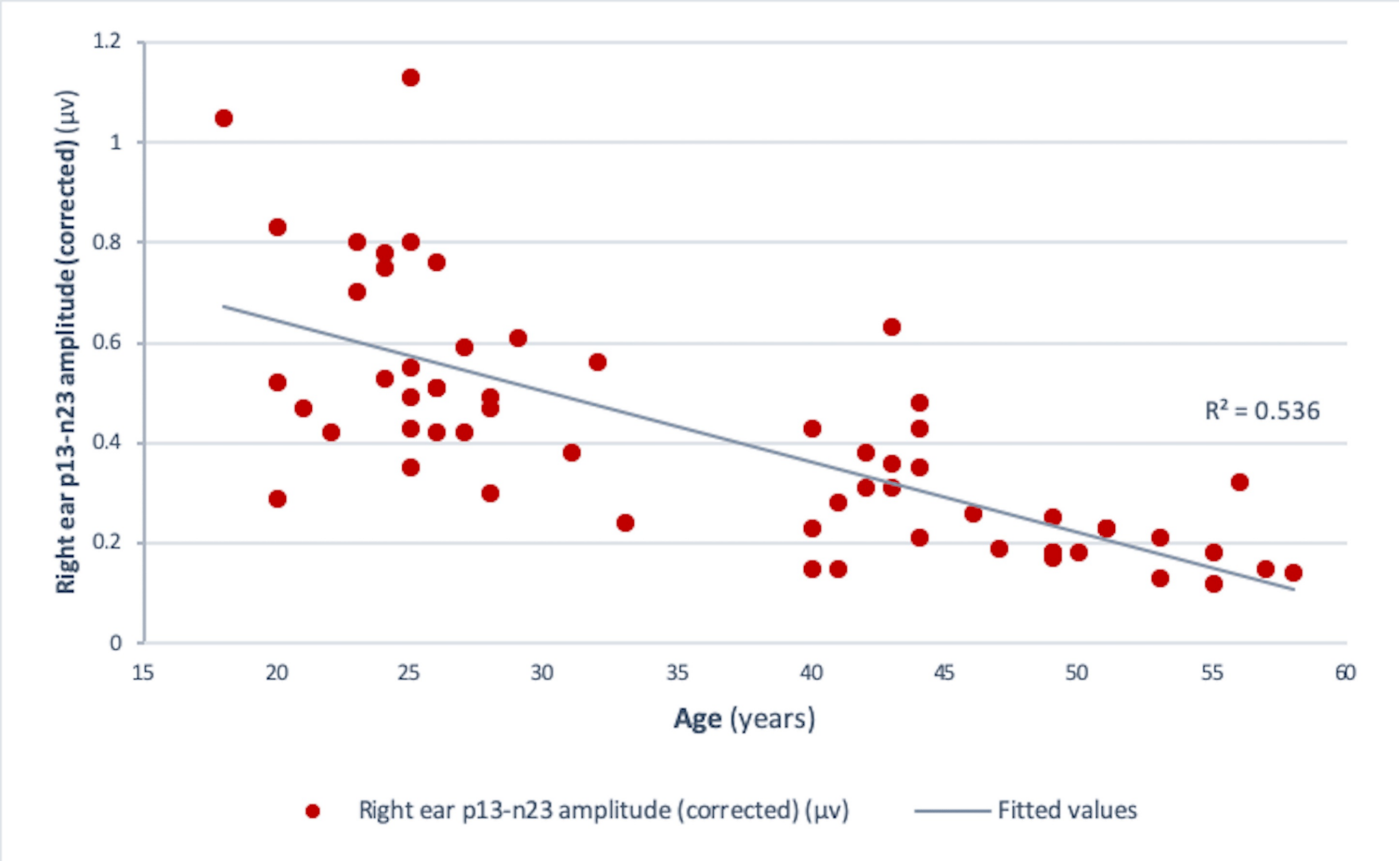
Scatter plot showing the relationship between age (in years) and right ear p13-n23 amplitude (corrected) (µv) Significant negative correlation observed between the age and right ear corrected amplitude (r = - 0.73, r² = 0.536, p<0.001) (Pearson correlation analysis) µv: microvolts

**Figure 7:**
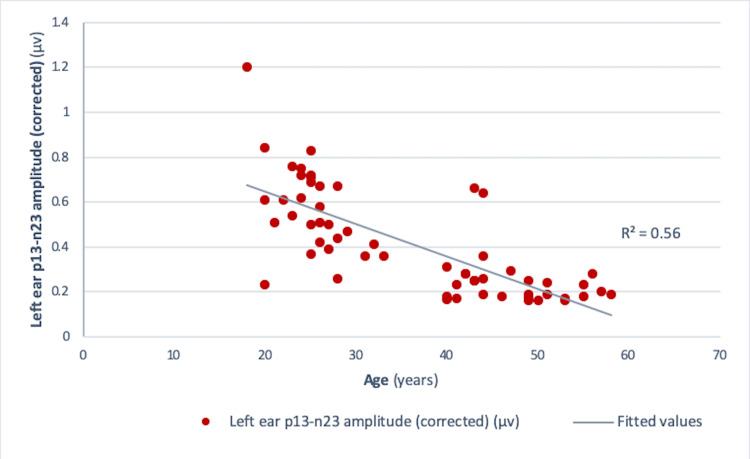
Scatter plot showing the relationship between age (in years) and left ear p13-n23 amplitude (corrected) (µv) Significant negative correlation observed between the age and left ear corrected amplitude (r = -0.75, r²=0.56, p = <0.001) (Pearson correlation analysis) µv: microvolts

**Figure 8:**
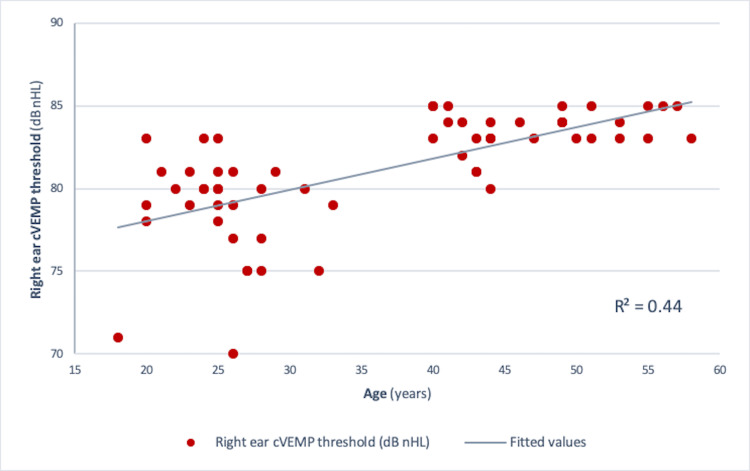
Scatter plot showing the relationship between age (in years) and right ear cVEMP threshold (dB nHL) Significant positive correlation was observed between the age of the participants and the stimulus threshold (r = 0.66, r² = 0.44, p<0.001) (Pearson correlation analysis) cVEMP: cervical vestibular-evoked myogenic potential; dB nHL: decibel normalized hearing level

**Figure 9:**
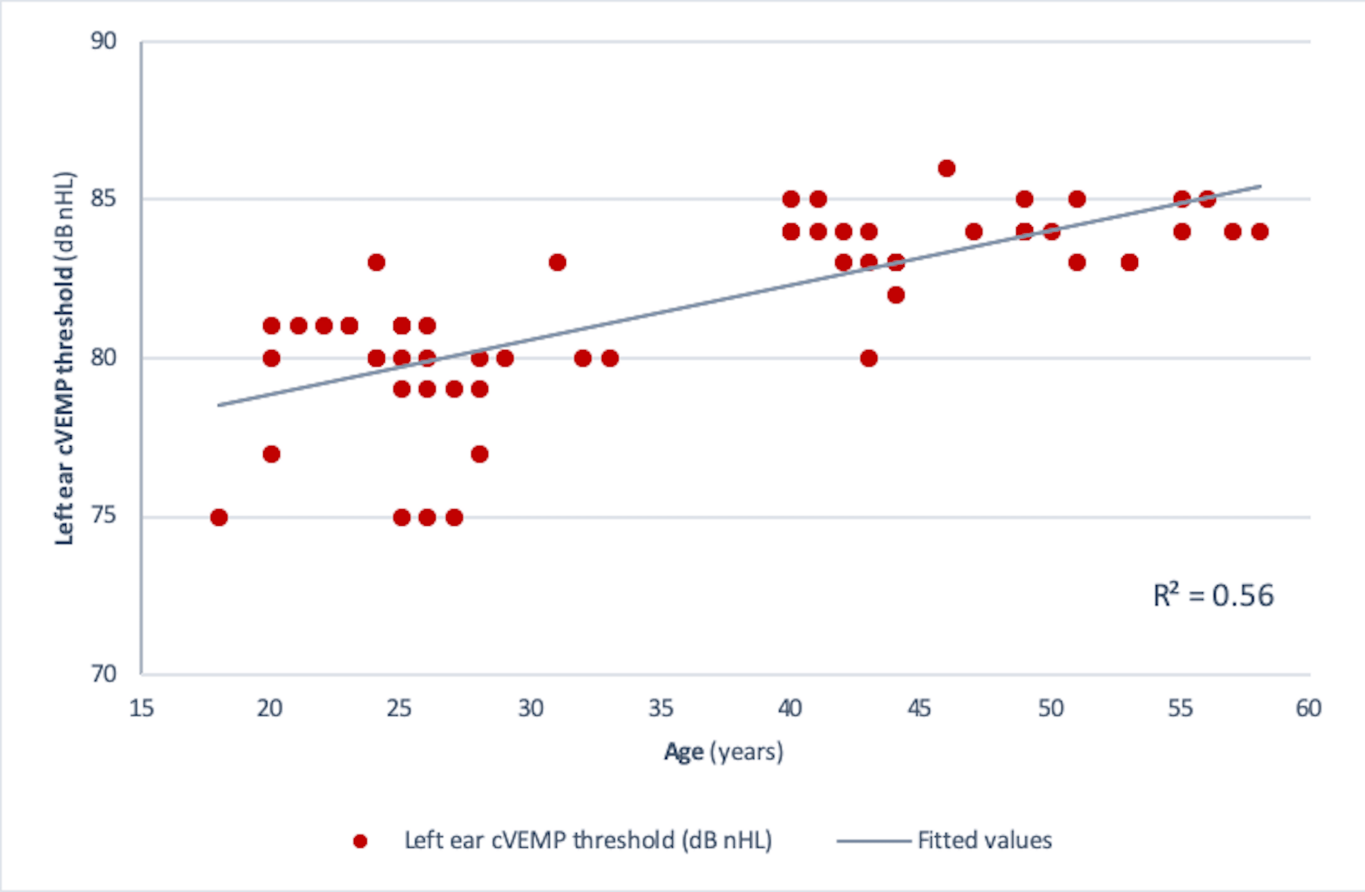
Scatter plot showing the relationship between age (in years) and left ear cVEMP threshold (dB nHL) Significant positive correlation was observed between the age of the participants and the stimulus threshold (r = 0.75, r² = 0.56, p<0.001) (Pearson correlation analysis) cVEMP: cervical vestibular-evoked myogenic potential; dB nHL: decibel normalized hearing level

Pearson correlation analysis revealed a significant negative correlation between age and amplitude in both the right ear (r = -0.73, r² = 0.534, p<0.001) and the left ear (r = -0.75, r² = 0.56, p<0.001) (Figures 4, 5). Similar results were obtained for those between age and corrected amplitude for the right ear (r = -0.73, r² = 0.536, p<0.001) and the left ear (r = -0.75, r² = 0.56, p<0.001) (Figures 6, 7). The results implied that as the age increased, the amplitude of cVEMP decreased statistically significantly. Conversely, a significant positive correlation was observed between age and thresholds in both the right ear (r = 0.66, r² = 0.44, p<0.001) and the left ear (r = 0.75, r² = 0.56, p<0.001) (Figures 8, 9). The above findings indicated that as the age increased, the thresholds for cVEMP increased, with a significant association. Gender comparisons did not demonstrate significant variations in cVEMP parameters (p>0.05) (Table 3). However, males exhibited a slight increase in amplitude asymmetry, which was statistically significant (p=0.018). But, the variation turned insignificant when the amplitude asymmetry for corrected amplitudes was compared (p>0.05) (Table 3).

**Table 3:** Comparison of cVEMP parameters between genders ^a^T-value (unpaired t-test). ^b^Chi-square value (Kruskal-Wallis test). ^*^P<0.05 cVEMP: cervical vestibular-evoked myogenic potentials; dB nHL: decibels normalized hearing level; IQR: interquartile range; ms: milliseconds; SD: standard deviation; µv: microvolts

cVEMP parameters	Gender	Values	t /chi^2^ value	P-value
Right ear p13 latency, ms, mean ±SD	Males	13.99 ±1.38	–0.96^a^	0.34
	Females	13.65 ±1.41		
Right ear n23 latency, ms, mean ±SD	Males	20.4 ±4.11	0.38^a^	0.70
	Females	22.86 ±1.68		
Left ear p13 latency, ms, mean ±SD	Males	13.63 ±1.21	0.15^a^	0.88
	Females	13.68 ±1.22		
Left ear n23 latency, ms, mean ±SD	Males	22.36 ±1.39	–0.07^a^	0.94
	Females	22.33 ±1.5		
Right ear uncorrected amplitude, µv, median (IQR)	Males	81.55 (44.1-146)	1.25^b^	0.26
	Females	76.3 (32.8-123)		
Right ear corrected amplitude, µv, median (IQR)	Males	0.37 (0.23-0.55)	0.002^b^	0.96
	Females	0.42 (0.23-0.52)		
Left ear uncorrected amplitude, µv, median (IQR)	Males	53.6 (38.1-145)	0.016^b^	0.90
	Females	70.45 (30.6-154)		
Left ear corrected amplitude, µv, median (IQR)	Males	0.28 (0.23-0.58)	0.07^b^	0.78
	Females	0.45 (0.2-0.61)		
Amplitude asymmetry (uncorrected), µv, median (IQR)	Males	0.09 (–0.05-0.21)	5.56^b^	0.018^*^
	Females	–0.01 (–0.13-0.08)		
Amplitude asymmetry (corrected), µv, median (IQR)	Males	0.05 (–0.06-0.09)	0.95^b^	0.33
	Females	–0.018 (–0.15-0.12)		
Right ear threshold, dB nHL, median (IQR)	Males	83 (79-84)	0.11^b^	0.7394
	Females	81 (80-83)		
Left ear threshold, dB nHL, median (IQR)	Males	83 (80-84)	0.17^b^	0.6843
	Females	81 (80-84)		
Threshold asymmetry, dB nHL, median (IQR)	Males	0 (–2-0)	0.12^b^	0.73
	Females	0 (–2-1)		

## Discussion

The present study intended to examine the major individual factors that influence cVEMP tests. We analyzed the impact of age and gender and ensured optimum and uniform SCM contraction level to minimize the influence of SCM contraction differences on cVEMP parameters under standard settings. The study investigated the pattern of various cVEMP parameters including response rates, amplitude, interaural amplitude asymmetry, threshold, threshold asymmetry and latency with respect to change in the above-mentioned individual features.

The detectability (response rates) of 500 Hz STB (95 dB nHL)-evoked cVEMPs was 100% for all the age groups in our study, with no absent cVEMP observed. Previous similar studies, however, have noted slightly lower response rates (86 %) with relatively similar stimulus conditions (500 Hz STBs, 98 dB nHL) and a similar age group studied [17]. Of note, 97-100% response rates have been found in some studies, but the stimulus intensities used were >98 dB nHL [18]. Regarding the age group, a major decline (62.5%) in the response rates has been reported for those 60 years old and above, with 95% detectability for subjects aged below 50 years old [12]. Our study reported absolute detection rates in the participants studied (≤60 years old) with the standard stimulus of 95 dB nHL, 500 Hz STBs recorded in a sitting position.

cVEMP amplitudes (peak to peak p13-n23) showed a decreasing trend with an increase in age (Figures 4-7). A significant diminution was found in the early and late middle ages [(35-44 years) and (45-60 years) (Groups 2 and 3)] with respect to young adults (18-35 years) (p<0.001) (Kruskal-Wallis test and post-hoc Dunn's test) (Table 1). The findings indicated that the amplitude decline started after the age of 35 years, which aligns with the previous studies [10,12]. The cVEMP thresholds showed an increase with a similar trend, consistent with age-related changes in VEMP thresholds reported by the previous studies (Figures 8, 9) [19, 20]. Threshold alterations with age have been linked with the broadening of vestibular tuning curves following neuronal changes due to aging [21]. The above findings pertain to the prevalence of vestibular dysfunction with increasing age. Age-related loss in the vestibular ganglion has been documented. The decrease in the number of ganglion cells has been reported to start after 30 years. Decrease in number, volume, and changes in shapes of otoconia both in the saccule and utricle have been supported by substantial evidence [19]. 

The reduction of interaural AA values computed with corrected amplitude as compared to those calculated with uncorrected amplitude, albeit small, was noticeable in all the age groups (Table 2) (Figures 1-3). This minimal incongruence observed may be attributable to the intricate EMG monitoring of SCM contractions during the recordings. The reduction, though statistically insignificant (p>0.05) (Wilcoxon signed-rank test), tended to minimize the interaural amplitude differences caused by SCM contraction levels, and the role has also been highlighted in previous research [9]. 

We did not find any statistically significant changes in p13 and n23 latencies with aging. Previous studies have also reported that aging did not change p13 or n23 latency [5,22]. Macambira et al. have reported that greater delay in latencies of cVEMP components was found in elderly people aged >60 years, which endorses our findings in adults ≤60 years [23]. We did not find any significant differences between genders for cVEMP parameters, which is consistent with the published results (Table 3) [12,14]. Amplitude responses have been suggested to be affected by the level of muscle activity. Various studies also point out that higher amplitude values in males than females may be caused by the greater level of muscle contraction in the former [24]. Previous research has highlighted the role of variance in muscle bulk between males and females in the gender differences found in oVEMP amplitude [25]. However, we could not investigate the role of differences in the muscle bulk/BMI among the genders, and its possible influence on cVEMP parameters. Nevertheless, the data for gender influences on cVEMP responses are conflicting in the previous literature. No explicit differences have yet been documented [12,14].

Limitations

This study has a few limitations. The impact of aging on VEMP responses could not be assessed in the age cohort older than 60 years. The vulnerable cVEMP parameters, including response rates, amplitude, and thresholds, could not be further assessed in the > 60-year-old participants. Gender comparisons incorporating BMI differences to further validate the evaluation could not be conducted. STB with a 500 Hz frequency was used to elicit cVEMP responses in the present study. The preferred range for AC stimulation in VEMP testing spans from 300 to 1000 Hz, and the influence of aging on frequency tuning in older adults has been reported. The frequency sensitivity (using different stimulus frequencies) in vestibular function could not be assessed.

## Conclusions

cVEMP was detectable with no loss of response noted for the age group (18-60 years) studied. However, in light of the remarkable age-related decline in cVEMP amplitude, the effective consideration of aging changes becomes crucial for the optimization of clinical evaluation. This could considerably improve the credibility of amplitude for cVEMP assessment. The threshold values should also be age-corrective for clinical interpretation. The present normative data in the adults (18-60 years) for cVEMP evoked with 500 Hz STB stimuli can be applied for diagnosing vestibular disorders in the regional clinical settings. The findings lend plausibility to the hypothesis that aging may impair saccular and related neural functions in terms of the cVEMP amplitude decline observed. cVEMP latencies, however, have a relatively modest impact on age-related deterioration in those aged ≤60 years. Further research on the individual factors, including the anatomical differences, ethnicity, and muscle bulk, would help gain deeper insights into the role of individual attributes in cVEMP interpretation.
